# Editorial: Application of the biogenic silver nanoparticles as antimicrobial and anticancer agents

**DOI:** 10.3389/fchem.2023.1180027

**Published:** 2023-03-10

**Authors:** Syed Rashel Kabir, Hamed Barabadi, Ehsan Karimi, Fatih Sen

**Affiliations:** ^1^ Department of Biochemistry and Molecular Biology, Faculty of Science, University of Rajshahi, Rajshahi, Bangladesh; ^2^ Department of Pharmaceutical Biotechnology, School of Pharmacy, Shahid Beheshti University of Medical Sciences, Tehran, Iran; ^3^ Department of Biology, Islamic Azad University, Mashhad, Iran; ^4^ Sen Research Group, Department of Biochemistry, Faculty of Art and Science, Kutahya Dumlupinar University, Kutahya, Türkiye

**Keywords:** silver nanoparticles, biological synthesis, antibacterial activity, antifungal activity, anticancer activity

Nanotechnology products have turned out to be more and more beneficial in biomedicine and have conducted the initiation of a mixed science identified as nanobiotechnology. Currently, nanobiotechnology provides the opportunity for ecofriendly-benign, fast, easy, and inexpensive synthesis of metal-based nanoparticles. Among different metallic nanoparticles, silver nanoparticles (AgNPs) have attracted significant interest owing to their unique physical, optical, chemical, and biological properties (Lee and Jun, 2019). Technology in this space is rapidly evolving. There are several biological approaches for the bioengineering of AgNPs involving phycosynthesis, phytosynthesis, and microbial synthesis (Almatroudi, 2020).

There are a large number of laboratory studies today on the biosynthesis of nanosized silver particles. This combinatorial research field is an advanced strategy for the safe production of nanomaterials. Deep knowledge in this field will allow researchers to explore innovative routes for the green synthesis of AgNPs. According to the literature, the researchers used herbal extracts, fungi, bacteria, and algae for the biosynthesis of AgNPs (Dawadi et al., 2021). The bioengineered AgNPs represented wide applications in biological and biomedical studies. Remarkably, the biogenic nanosized silver particles exhibited significant antineoplastic and antimicrobial properties (Algotiml et al., 2022; Alduraihem et al., 2023). Gathering knowledge in this subject helps the researcher to find novel anticancer and antimicrobial nanomedicines faster. Hence, this Research Topic mainly focuses on the recent achievements of the application of biogenic nanosized silver particles as antimicrobial and anticancer agents.

Gur reported the herbal-mediated synthesis of AgNPs by using the aqueous fruit extract of sumac (*Rhus coriaria* L.) with spherical morphology and an average particle size of 4 nm. Then, the antibacterial and antifungal performance of biosynthesized AgNPs were evaluated against Gram-positive bacteria (*Bacillus cereus*, *Bacillus subtilis*, *Staphylococcus aureus*, and *Enterococcus faecalis*), Gram-negative bacteria (*Escherichia coli* and *Pseudomonas aeruginos*) and *Candida albicans* fungus by disk diffusion assay. The results showed that the biogenic AgNPs did not show antibacterial activity against *S. aureus* and *E. coli*. However, the bioengineered nanoparticles exhibited antimicrobial activity against *B. cereus*, *B. subtilis*, *E. faecalis*, *P. aeruginos*, and *C. albicans* with inhibition zone (IZ) of 11 ± 1.2, 14 ± 0.5, 10 ± 0.7, 10 ± 0.68, and 12 ± 0.2 mm, respectively. In the next step, an agarose gel electrophoresis assay was employed to study the DNA damage inhibition activity of *R. coriaria*-derived AgNPs. The results showed that the biogenic AgNPs at the concentration of 1,000 μg·mL^−1^ had a good protective effect on pBR322 plasmid DNA. In a study, Bold et al. reported the plant-mediated synthesis of AgNPs by using the aqueous root extract of *Rhodiola rosea* with spherical morphology and an average particle size of 20 nm. In this study, the biosynthesized AgNPs showed not only considerable antibacterial and antioxidant properties, but also significant wound-healing activity in an AgNP-loaded ointment formulation on burn injury in the BALB/c mice model. Compared to untreated burn wounds, the AgNP-loaded ointment not only reduced the wound size and mast cell migration but also lowered the epidermis layer. Moreover, Mussin and Giusiano reviewed recent advances in the antifungal performance of biosynthesized AgNPs. The authors reported that the minimal inhibitory concentration (MIC) of AgNPs against various fungal species was in a range of 0.002–315.5 μg·mL^−1^. The authors reported synergistic antifungal properties of AgNPs with standard antifungal agents, such as ketoconazole, clotrimazole, itraconazole, fluconazole, etc. The authors also stated that due to the difference in the biological capping agents in the biosynthesis of AgNPs, the safety of these nanostructures should be evaluated individually. Furthermore, Dugganaboyana et al. reported the green fabrication of spherical AgNPs by employing the aqueous root extract of *Salacia oblonga* with a size distribution of less than 99.8 nm. The phytofabricated AgNPs not only exhibited significant antioxidant and antibacterial activities but also showed considerable carbohydrate-hydrolyzing enzyme alpha-amylase inhibitory potential with a half maximal effective concentration (EC_50_) of 58.38 μg·mL^−1^.

In the future, biofabricated AgNPs will continue to play a vital role in nanomedicine. However, further investigations are required to deeply study the pharmacokinetics and pharmacodynamics of these nanomaterials in an animal model. Moreover, the safety of these nanoparticles should be widely studied before the translation of the *in vitro* studies to clinical investigations.
